# Correction: Performance evaluation of interfacial polymerisation-fabricated aquaporin-based biomimetic membranes in forward osmosis

**DOI:** 10.1039/c9ra90045d

**Published:** 2019-06-18

**Authors:** Zhixia Liang, Yanbin Yun, Manxiang Wang, Guicheng Liu, Peng Lu, Woochul Yang, Chunli Li

**Affiliations:** School of Environmental Science and Engineering, Beijing Forestry University Beijing 100083 China yunyanbin@bjfu.edu.cn; Center for Energy Storage Research, Clean Energy Institute, Korea Institute of Science and Technology (KIST) Hwarang-ro 14-gil 5, Seongbuk-gu Seoul 02792 Republic of Korea wmx8866@163.com; Department of Physics, Dongguk University Seoul 04620 Republic of Korea; College of Material Science and Chemical Engineering, Ningbo University Zhejiang 315211 China; New Technique Centre, Institute of Microbiology, Chinese Academy of Sciences Beijing 100101 China

## Abstract

Correction for ‘Performance evaluation of interfacial polymerisation-fabricated aquaporin-based biomimetic membranes in forward osmosis’ by Zhixia Liang *et al.*, *RSC Adv.*, 2019, **9**, 10715–10726.

The authors regret that affiliation e was missing in the original article. In addition, the institute name in affiliation b was incorrect. The correct affiliations are as presented here.

The Royal Society of Chemistry apologises for these errors and any consequent inconvenience to authors and readers.

## Supplementary Material

